# Genomic and phenotypic analyses reveal *Paenibacillus polymyxa* PJH16 is a potential biocontrol agent against cucumber fusarium wilt

**DOI:** 10.3389/fmicb.2024.1359263

**Published:** 2024-03-25

**Authors:** Fan Yang, Huayan Jiang, Kai Ma, Abeer Hegazy, Xin Wang, Shen Liang, Gaozheng Chang, Liqin Yu, Baoming Tian, Xuanjie Shi

**Affiliations:** ^1^Institute of Horticulture, Henan Academy of Agricultural Sciences, Graduate T&R Base of Zhengzhou University, Zhengzhou, Henan, China; ^2^School of Agricultural Sciences, Zhengzhou University, Zhengzhou, China; ^3^National Water Research Center, Shubra El Kheima, Egypt; ^4^Henan Natural Products Biotechnology Co., Ltd., Zhengzhou, China

**Keywords:** *Fusarium oxysporum* f.sp. *cucumerinum*, *Paenibacillus polymyxa*, biocontrol agent, genome sequencing and assembly, biocontrol mechanism

## Abstract

In recent years, bacterial-based biocontrol agents (BCA) have become a new trend for the control of fungal diseases such as fusarium wilt that seriously threaten the yield and quality of cucumber, which are transmitted through infested soil and water. This study was set out with the aim of figuring the mechanism of the isolated rhizobacterial strain *Paenibacillus polymyxa* PJH16 in preventing *Fusarium oxysporum* f. sp. *cucumerinum* (*Foc*). Biocontrol and growth-promoting experiments revealed that bacterial strain causes effective inhibition of the fungal disease through a significant growth-promoting ability of plants, and had activities of β-1,3-glucanase, cellulase, amylase and protease. It could produce siderophore and indole-3-acetic acid, too. Using the high-throughput sequencing tool PacBio Sequel II system and the database annotation, the bacterial strain was identified as *P. polymyxa* PJH16 and contained genes encoding for presence of biofilm formation, antimicrobial peptides, siderophores and hydrolyases. From comparing data between the whole genome of *P. polymyxa* PJH16 with four closely related *P. polymyxa* strains, findings revealed markedly the subtle differences in their genome sequences and proposed new antifungal substances present in *P. polymyxa* PJH16. Therefore, *P. polymyxa* PJH16 could be utilized in bioengineering a microbial formulation for application as biocontrol agent and bio-stimulant, in the future.

## 1 Introduction

Cucumber is an important economic crop. China is the main producer of cucumber, and its planting area and scale have ranked first in the world for many years. One cucumber disease that poses a serious threat to yield and quality is cucumber fusarium wilt caused by the fungus *Fusarium oxysporum* f. sp. *cucumerinum* which belongs to Ascomycota (Gao et al., 2014). This fungus is a soil-borne pathogen that can infect plants at any stage of growth. It begins invading the wounds and root hair growth points, then colonizes the vascular bundles resulting in blocked absorption of water and nutrients for plants, and ultimately leads to plant wilting and death. Due to the disease damaging effect and difficulty to control, it has limited greatly the development of cucumber global industry (Zhou et al., 2017).

To combat the phytopathogen, chemical fungicides are usually used which may cause environmental and health concerns, as well as the problem of pathogen resistance. Biological control is a powerful option for treating bacterial and fungal infections. Therefore, microbial biocontrol agents (BCA) have been developed over the past few decades such as employing biocontrol bacteria to manage plant diseases, decrease the reliance on chemical pesticides and promote sustainable agriculture (Maciag et al., 2023). At present, many bacteria from different genera have been identified as biocontrol agents, including *Bacillus*, *Paenibacillus*, *Agrobacterium*, *Bradyrhizobium*, *Acinetobacter*, *Azospirillum*, *Azotobacter*, *Pseudomonas*, *Rhizobium*, and *Streptomyces* (Ayaz et al., 2021). Since *Paenibacillus polymyxa* belongs to the genus *Paenibacillus* of the family Bacillaceae. It is a Gram-positive bacteria widely distributed in nature, non-pathogenic to plants, their cell wall does not contain endotoxins, and they are harmless to humans and animals. Accordingly, it is an ideal bacteria to use as a biocontrol agent. Applying BCA to crops allows them to biologically suppress plant diseases through variety of modes of action (Köhl et al., 2019). BCA may directly interact with the pathogen through antibiosis or hyperparasitism. Hyperparasites infiltrate and destroy bacterial and fungal pathogen. Without directly engaging in antagonistic interactions with the pathogen, they can either improve or produce resistance against phytopathogen (Conrath et al., 2015). From that point, part of plant defense systems is to enhance resistance by producing reactive oxygen species, phytoalexins, phenolic compounds, pathogenesis-related proteins, and physical barriers including modifying cell walls and cuticles (Wiesel et al., 2014). Another direct form of action is the production of secondary metabolites that have antagonistic effects on a range of phytopathogen (Ghorbanpour et al., 2018), and are commonly including ribosomal peptides (RPs), non-ribosomal peptides (NRPs), antibiotics, polyketides (PKs), and volatile organic compounds (VOCs) (Farzand et al., 2019a). Surfactin, iturin, mixirin, and fengycin are examples of lipopeptides with antifungal properties. Besides, the formation of biofilms reduces the number of pathogens. Extracellular enzymes like chitosanase, protease, glucanase, and cellulase are produced by *Bacillus* species, which are known to adhere to mycelial cell walls and deform hyphae (Farzand et al., 2019b).

In recent years, the rapid development of sequencing technology has becomeincreasingly more efficient, providing a major key to in-depth study of the function and mechanism of bacteria in disease control. Sequencing efforts help to understand the gene function or are used to design gene engineering experiments. Additionally, annotation in the same species can act as a template for reading maps, and can be aligned and compared to study molecular evolution (Giani et al., 2020). There are few studies on the complete genome sequence of this bacterial strain and other on its ability to regulate phytopathogens through the processes of antagonistic activity (production of antibiotics), colonization (biofilm formation), and induced resistance (Luo et al., 2018).

This study focused on a bacterial strain named *Paenibacillus polymyxa* PJH16 isolated from the rhizosphere soil of healthy cucumber plants, and investigates its inhibitory effect on the fungus disease *Fusarium oxysporum* f. sp. *cucumerinum in vitro* and *in vivo*. To elucidate its mechanisms in promoting cucumber plant growth and controlling fusarium wilt, 16S rRNA gene sequence phylogenetic tree and average nucleotide identity (ANI) analysis are investigated.

## 2 Materials and methods

### 2.1 Isolation of bacteria and screening of soil antagonistic bacteria

Through the application of dilution plate methodology, we successfully extracted the bacterial strain *P. polymyxa* PJH16 from the soil within the cucumber cultivation region of Beiwang Village, Luolong District, Luoyang City, Henan Province, China—a region known for being a high incidence area of cucumber fusarium wilt. The bacterial strain *P. polymyxa* PJH16 was nurtured on Luria Bertani medium (LB) plates. Simultaneously, the *Fusarium oxysporum* f. sp. *cucumerinum* FJH36 (*Foc* FJH36) fungi were isolated and conserved at the Institute of Horticulture, Henan Academy of Agricultural Sciences (Yang et al., 2023).

As for Antagonistic bacterial screening, the plate confrontation culture method was used with *Foc* FJH36 fungi as indicator fungi. A puncher with a diameter of 5 mm was used to punch the pathogenic fungi cake at the edge of the *Foc* plate into the center of the Potato Dextrose Agar (PDA) plate, and the bacteria were inoculated at a distance of 25 mm from the bacteria cake, which was located in the same line as the midpoint of the bacteria cake. The control was a PDA plate inoculated with *Foc* fungi in the absence of bacteria. Triplicate plates were inoculated at 30°C for 7 days. The fungal growth inhibition was assessed in fungi co-cultured with bacteria in comparison with control plates (Xu et al., 2020).

### 2.2 Observation of antagonistic mycelium

The mycelium of *Foc* FJH36 in the antagonistic plate was cut, and the volume of the sample was not more than 3 mm^3^. After the sample being gently rinsed with phosphate buffer saline (PBS) (2.7 mM KCl, 2.0 mM KH_2_PO_4_, 137 mM NaCl, 10 mM Na_2_HPO_4_, pH 7.4), it was quickly placed into the electron microscope fixative at room temperature for 2 h, then transferred to 4°C preservation. The fixed sample was rinsed three times for 15 min each time with 0.1 M phosphate buffer PB (2.7 mM KCl, 2.0 mM KH_2_PO_4_, 10 mM Na_2_HPO_4_, pH 7.4). A 0.1 M phosphate buffer PB (pH 7.4) was used to prepare 1% osmic acid, and the samples were fixed at room temperature in the dark for 2 h. Rinse three times for 15 min each with 0.1 M phosphate buffer PB (pH 7.4). The tissue was subjected to gradient dehydration in alcohol concentrations of 30, 50, 70, 80, 90, 95, 100% for 15 min each time, followed by 15 min soak in isoamyl alcohol. For drying, the samples were placed in a Quorum K850 critical point dryer. To examine and take photographs, the samples were tightly adhered to the double-sided adhesive of the conductive carbon film and placed on the sample table of the Hitachi MC1000 ion sputtering instrument for gold spraying for 30 s, and Hitachi SU8100 scanning electron microscope (Hitachi Higher Technologies Corp., Tokyo, Japan) was employed.

### 2.3 Biocontrol effect of PJH16 on cucumber fusarium wilt

To evaluate the biocontrol effect of bacteria on fungal disease, the pot experiment was carried out. The first step in this process was to prepare suspensions of *Foc* FJH36 fungi and bacterial strain PJH16, and germinating the seeds of cucumber as the following:

After growing *Foc* FJH36 on a PDA plate for 5 days at 30°C, ten fungal cakes were taken from the edge of the pathogen using a 5 mm puncher and cultured in a 100 mL Potato Dextrose Broth (PDB). The cultures were shaken for 3 days at 30°C, 180 rpm, and then the filtered cultures using 4 layers of sterile gauze were diluted with sterile water to a volume of 1 × 10^6^ spores/mL pathogen spore suspension. For the bacterial strain PJH16, it was cultured in LB medium at 37°C, 180 rpm for 24 h, centrifuged at 4000 xg for 5 min, and diluted to 1 × 10^8^ CFU/mL with LB medium.

**Figure 1:**
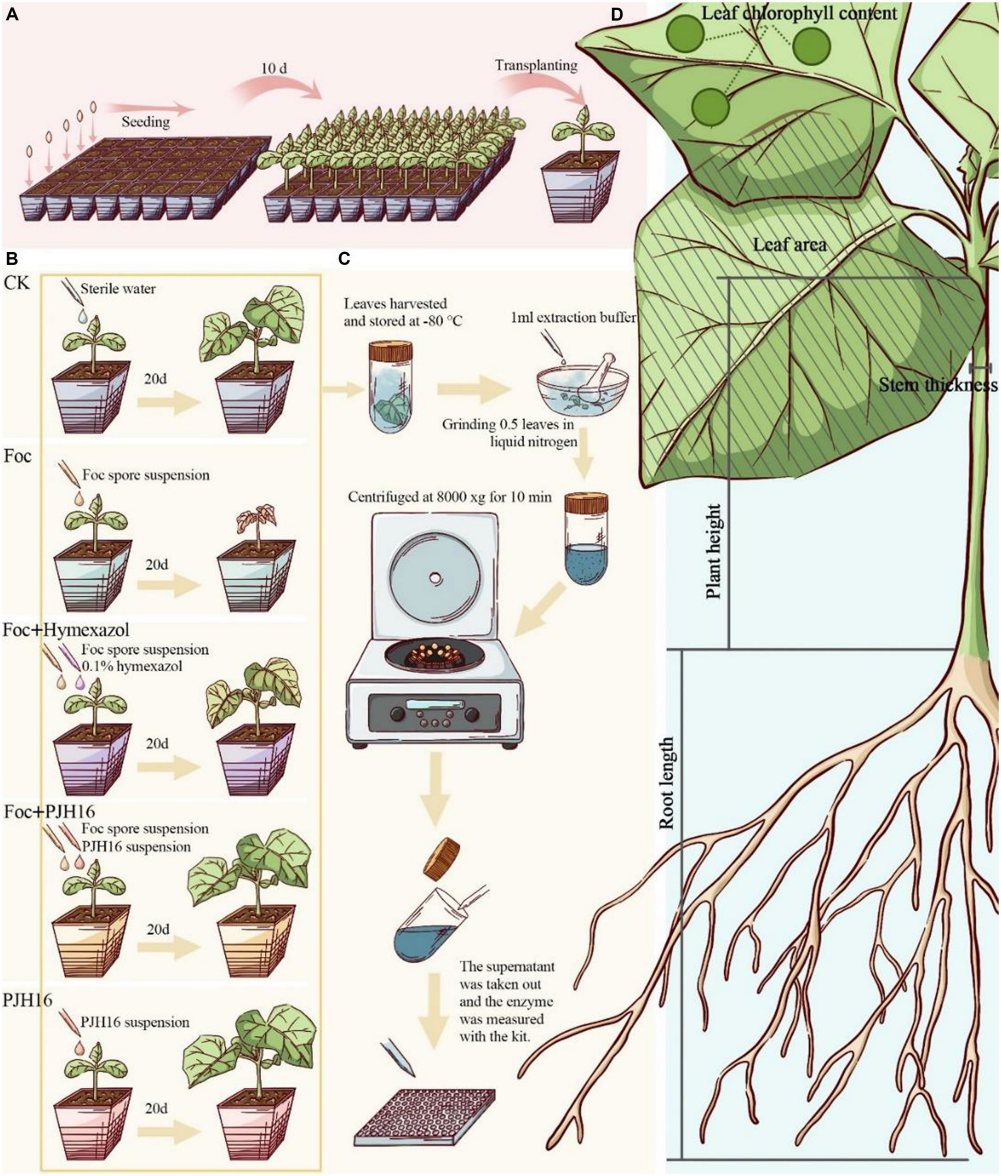
Experimental flow for this study. **(A)** Plug seedling, and transferred to the pot after 10 days; **(B)** procedure for the pot experiment; **(C)** after 20 days of inoculation, the activity of defense enzymes in cucumber leaves was determined; **(D)** the data of cucumber seedlings were measured 40 days after inoculation.

For seed germination, cucumber seeds of the Bojie 616 variety, characterized by their robust, full, and uniform size, were carefully chosen. Initially, the cucumber seeds underwent a series of steps: they were immersed in 75% ethanol (v/v) for 30 s, rinsed five times with sterile water, and soaked in 55°C warm water for 10 min. Subsequently, the seeds were soaked in distilled water for 4 h, and gauze moistened with sterile water was utilized. Following thorough rinsing, the seeds were neatly arranged on the gauze and placed in a constant temperature incubator at 28°C. White cucumber seeds were then sown in a seedling plug (5 × 8) with sterile matrix for a duration of 10 days. Once the seeds had developed into seedlings, two-leaf seedlings of uniform size were selected and transplanted into pots with a diameter and height of 10 cm, along with 400 g of sterile matrix (Figure 1A).

Subsequent to the initial phase, this study incorporated four treatments and a control for the seedlings. Watering was performed once every two days initially, followed by the continued use of only sterile distilled water throughout the growth period. The treatments were implemented as outlined in Figure 1B.

For the experimental setup, five treatments with distinct procedures were employed, each with five replicates, and five pots for each replicate, totaling 125 pots. The greenhouse conditions maintained an ambient temperature ranging from 14 to 25°C, with a photoperiodic lighting schedule of 16 h of light followed by 8 h of darkness.

We measured plant height, stem diameter, leaf area, chlorophyll content, shoot fresh weight, root fresh weight, shoot dry weight and root dry weight on the 20th and 40th days during the whole growth period (Yang et al., 2023; Figure 1D).

Finally, in order to come into view of the biocontrol effect of PJH16 on cucumber fusarium wilt, several formulas were calculated such as: Strong Seedling index, Disease severity, Disease Index (DI) and Disease Control efficacy (Chen H. et al., 2010; Chen F. et al., 2010). For the strong seedling index, it was calculated with the following formula: Strong seedling index = (stem diameter/plant height + root dry weight/shoot dry weight) × whole plant dry weight. As for estimation of the disease severity and disease indices of plants, they were recorded at 40 days. Disease severity was measured by the assessment of disease symptoms of leaves, that divided into 5 levels: level 0, leaves without symptoms; level 1 grade, and the leaf wilting was lower than 1/4 of cucumber seedlings; level 2, 1/4–1/2 of cucumber seedling leaves wilting; level 3, more than 1/2 leaves of cucumber seedlings wilted; level 4, the whole cucumber plant withered and died. The disease index was calculated using DI = [[(0 × N0) + (1 × N1) + (2 × N2) + (3 × N3) + (4 × N4)]/T × 4] × 100, where N is the number of cucumber seedlings scored for each disease, and T is the total number of cucumber seedlings. Incidence = [N1 + N2 + N3 + N4]/T × 100%. The disease control efficacy was figured through the next calculation: Control efficacy = (control DI-treatment DI)/control DI × 100%.

### 2.4 Determination of defense enzyme activity in cucumber leaves

As shown in Figure 1C, on the 20th day after inoculation, plant leaves were collected for determination of enzyme activity. Polyphenol oxidase (PPO), superoxide dismutase (SOD), catalase (CAT), phenylalanine ammonia-lyase (PAL) and lipoxidase (LOX) detection kits were used to determine the enzyme activity according to the manufacturer ‘s (Solarbio, Beijing, China) guidelines. The absorbance was measured by a microplate reader (Tecan Spark, Tecan, Switzerland).

### 2.5 Detection of plant growth-promoting bacteria and enzymatic activity

In order to evaluate the plant growth-promoting and biocontrol characteristics of strain PJH16, we referred to the previous research methods (Yang et al., 2023) to detect the β-glucanase activity, amylase activity, siderophore production ability, cellulase activity, protease activity and IAA production ability of strain PJH16.

Single colony of PJH16 strain was inoculated in β-glucan agar medium (0.5 g yeast extract, 1.0 g peptone, 0.05 g glucose, 0.5 g NaCl, 0.01 g Congo red and 18.0 g agar/L, pH 7.0), and cultured in an incubator at 30°C for 48 h. A clear area around the flora was observed, indicating that the strain had β-glucanase activity.

Single colony of PJH16 strain were inoculated in starch agar medium (10.0 g soluble starch, 10.0 g tryptone, 5.0 g glucose, 5.0 g NaCl, 5.0 g beef extract and 18.0 g agar/L, pH 7.0), and cultured in an incubator at 30°C for 48 h. Lugol ‘s iodine solution (1% iodine in 2% potassium iodide w/v) was added to the starch agar medium to make it evenly spread on a flat plate. After standing for 1 min, a colorless halo around the colony was observed, indicating that the strain had amylase activity.

Single colony of PJH16 strain was inoculated in Chrome Azurol S blue agar medium (10.0 mL 20% sucrose solution, 30.0 mL 10% acid hydrolyzed casein, 1.0 mL 1.0 mmol/L CaCl_2_, 5.0 mL 0.1 mol/L phosphate buffered saline (pH 6.8), 50.0 mL CAS staining solution and 18.0 g agar/L, pH 7.0), and cultured in an incubator at 30°C for 48 h. The surrounding of the colony changed from blue to orange, indicating that the strain could produce siderophores.

Single colony of PJH16 were inoculated in carboxymethyl cellulose agar medium (5.0 g CMC-Na, 0.1 g MgSO_4_·7H_2_O, 0.25 g K_2_HPO_4_, and 18.0 g agar/L, pH 5.5) and cultured at 30°C for 48 h. After staining with 1% (m/v) Congo red stain, the medium was washed with sterile distilled water. If there is a transparent halo around the colony, it indicates that the strain can produce cellulase.

The single colony of PJH16 strain was inoculated in skim milk agar medium (0.1 g CaCl_2_, 5.0 g NaCl, 10.0 g peptone and 18.0 g agar/L, pH 7.0), and cultured in an incubator at 30°C for 48 h. If a transparent area was present around the colony, it indicated that the strain had protease activity.

Single colonies of PJH16 were inoculated in L-tryptophan nutrient broth (3.0 g beef extract, 10.0 g peptone, 5.0 g NaCl, 0.5 g L-tryptophan/L, pH 7.2) and cultured in an incubator at 30°C for 48 h. After centrifugation at 14,000 xg for 10 min, 1 mL supernatant was mixed with 2 mL Salkowski dye, and then placed in dark at room temperature for 30 min. The color changed from yellow to orange, indicating the production of IAA.

### 2.6 DNA extraction, genome sequencing, and assembly of strain PJH16

In order to obtain the genomic information of strain PJH16, the single colony of strain PJH16 was first inoculated in LB medium and cultured for 18 h at 37°C and 150 rpm. Subsequently, according to the manufacturer’s instructions, genomic DNA was extracted using the Mini BEST bacterial genomic DNA extraction kit Ver.3.0. In order to construct an insertion sequencing library, about 10 kb DNA insertion fragments were prepared and sequenced using the PacBio Sequel II system (Pacific Biosciences, Menlo Park, CA, United States) of Frasergen (Wuhan, Hubei, China). The sequencing reads were assembled from scratch using HGAP4 (Chin et al., 2013) and Canu (v.1.6) (Koren et al., 2017) software, and the coverage depth of the genome was analyzed by comparison tools. Finally, the complete genome sequence of the assembled strain PJH16 was stored in NCBI GenBank, and the accession number was CP137742. In addition, a circular map of the genome of strain PJH16 was constructed by using Circos (Krzywinski et al., 2009).

### 2.7 Genome annotation of strain PJH16

The annotation of the PJH16 strain genome was conducted using Glimmer (v3.02) (Delcher et al., 2007). Identification of tRNA and rRNA genes was performed using tRNAscan-SE (v2.0) (Lowe and Eddy, 1997) and RNAmmer (v1.2) (Lagesen et al., 2007), respectively. BLASTx analysis utilized the NCBI non-redundant protein database (NR), SwissProt, Orthologous Group (COG), Kyoto Encyclopedia of Genes and Genomes (KEGG), and Gene Ontology (GO). The blastp command of diamond (v2.0.9.147) software was employed to align the protein sequences of predicted genes with the NR microbial library (20210213 download version), using an E value of 1e^−5^, and the hit with the highest score was chosen as the final annotation.

The same diamond blastp command was applied to align the predicted gene protein sequences with the SwissProt (v2021_03) library, COG2020 library, and the full library of KOBAS-v3.0 (Xie et al., 2011), each with an E value of 1e^−5^. The hits with the highest scores were selected as the final annotation results. For GO annotation, SwissProt annotation results were mapped by ID Mapping (20210616), and go-basic.obo (v2021-09-01) was used for hierarchical annotations.

Specific functional annotation modules were constructed by integrating various function-related databases and annotation methods. These modules included drug resistance genes (CARD), pathogenic virulence factors (VFDB), and membrane transporter classification (TCDB). The predicted gene protein sequences were aligned to the CARD-v3.1.4 library, the SetA library of pathogenic virulence factors (VFDB, Last updated: Sep 24, 2021), and the TCDB library using diamond blastp with an E value of 1e^−5^. The hits with the highest scores were selected as the final annotation results based on the predicted gene amino acid sequence file.

### 2.8 Analysis and identification of strain PJH16

The 16S rRNA gene sequence tree of 114 strains (Supplementary Table S1) based on strain PJH16 and 20 strains of *P. terrae*, 15 strains of *P. polymyxa*, 23 strains of *P. xylanexedens*, 14 strains of *P. thiaminolyticus*, 11 strains of *B. subtilis*, 12 strains of *B. mycoides* and 19 strains of *B. cereus* was constructed by MEGA 7.0 using Neighbor Joining method (Kumar et al., 2016). The average nucleotide identity (ANI) of 10 strains of *P. polymyxa* and 3 strains of *B. mycoides* with PJH16 was calculated using the ANI calculator (Yoon et al., 2017).

### 2.9 Analysis of secondary metabolic genes in CAZymes of strain PJH16

During the genome analysis of strain PJH16, we employed dbCAN2 (Zheng et al., 2023) and HMMER (v3.1b2) (Finn et al., 2011) to align the predicted protein sequences with the Carbohydrate Active Enzyme (CAZy) database (Lombard et al., 2014), with a set E value threshold of 1e^−15^. Additionally, utilizing antiSMASH version 7.0.0, we conducted a comparative analysis of the protein-coding genes in the genome of strain PJH16 against the CAZy database to pinpoint gene clusters associated with the synthesis of secondary metabolites (Blin et al., 2019). Secondary metabolites are generally controlled by multiple genes, and their coding genes are usually clustered in the genome. This gene cluster is the secondary metabolite synthesis gene cluster (BGC).

### 2.10 The collinearity analysis of strain PJH16

We used Mauve (v2.3.1) (Darling et al., 2004) to globally align the chromosomes of *P. polymyxa* PJH16, *P. polymyxa* HY96-2, *P. polymyxa* SQR-21, *P. polymyxa* CF05 and *P. polymyxa* DSM 36. In order to visualize the alignment results, we used TBtools (v1.0697) (Chen C. et al., 2020) to draw the collinearity diagram of PJH16 and four *P. polymyxa* strains.

### 2.11 Statistical analysis

We used SPSS v21.0 for statistical analysis by one-way analysis of variance (ANOVA). The mean was compared with Duncan’s multi-range test with a probability of *p* ≤ 0.05.

## 3 Results

### 3.1 Antagonistic characteristics of PJH16 strain

**Figure 2:**
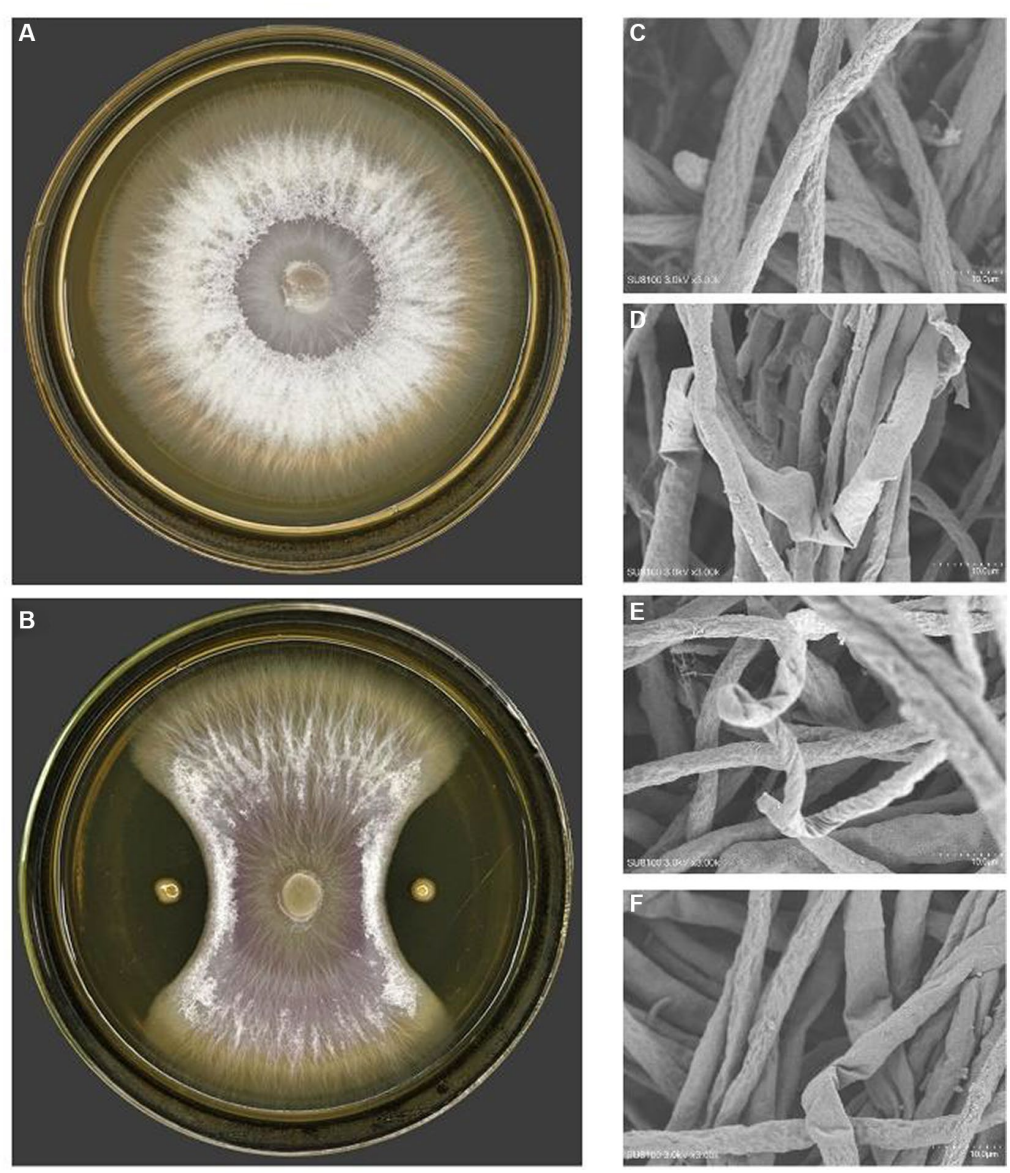
The antagonistic effect of PJH16 on *Foc* FJH36. **(A)** The colony morphology of *Foc* FJH36 without PJH16 strain on PDA plate; **(B)** the colony morphology of *Foc* FJH36 containing PJH16 strain on PDA plate; **(C)** *Foc* FJH36 normal development of mycelial morphology; **(D)** PJH16 strain caused the mycelium of *Foc* FJH36 to fold; the **(E)** PJH16 strain caused the mycelium of *Foc* FJH36 to distort; **(F)** PJH16 strain caused the mycelium of *Foc* FJH36 to shrink.

On the purpose of figuring out the antagonistic activity against phytopathogen of the genus *Fusarium*, it was carried out the antagonistic bacterial screening for PJH16 strain isolated from soil of cucumber planting area with high incidence of cucumber fusarium wilt. It has been illustrated that the inhibition rate of PJH16 was 88.36%, with antagonistic band width was 11 mm (Figures 2A,B). At the same time, it was verified by scanning electron microscopy and observed the morphology of the mycelium of the antagonistic *Fusarium oxysporum* showed abnormal morphology, such as folding (Figure 2D), twisting (Figure 2E) and shrinkage (Figure 2F), comparing with the mycelium of the control treatment that was smooth and no abnormal changes (Figure 2C). Thus, the *Paenibacillus polymyxa* PJH16 strain presented anatogonistic behavior when paired with the genus *Fusarium oxysporum* f. sp. *cucumerinum Foc* FJH36, affecting the development pattern.

### 3.2 Anti-fusarium wilt activity of PJH16 strain *in vivo*

The experiment of phytopathogenic effect *in vivo* under controlled condition is a well-established approach in highlighting the anti-fusarium wilt activity. For such a goal, four treatments for cucumber seedlings were compared with control to assess the most effective anti-disease treatment. The first treatment was to evaluate the effect of disease alone on the seedlings; the second to estimate the fungicidal effect, the third to determine whether the bacterial strain can reduce the disease effect; and the fourth one with only the isolated bacterial strain to evaluate its influence. Afterwards, the severity of disease, disease incidence, disease index and control efficacy were determined for cucumber seedlings.

It is noteworthy that the severity of fusarium wilt in cucumber seedlings treated with PJH16 decreased 20 days after *Foc* inoculation. Furthermore, at 40 days, the disease incidence, disease index, and control efficacy of the three treatments indicated that the PJH16 strain significantly alleviated the wilt symptoms caused by *Foc*, with a reduction of 98.08% in disease incidence and 92.89% in disease index. Notably, PJH16 exhibited a control efficacy against *Fusarium oxysporum* higher than that of the chemical fungicide Hymexazol (92.71, 88.83%) (Table 1).

**Table 1:** The disease incidence, disease index, and control efficacy of hymexazol and *Paenibacillus polymyxa* PJH16 against cucumber wilt disease.

Treatment	Disease incidence (%)	Disease index	Control efficacy (%)
*Foc*	95.69 ± 0.29^a^	121.26 ± 0.31^a^	
*Foc* + 0.1% Hymexazol	6.98 ± 0.35^b^	13.55 ± 0.21^b^	89.02 ± 0.33^a^
*Foc* + PJH16	1.84 ± 0.12^c^	8.62 ± 0.08^c^	94.00 ± 0.16^b^

The data are presented as mean ± standard deviation (SD). Within the same column, different letters (a–c) indicate significant differences at *p* < 0.05 level.

### 3.3 Effects of PJH16 strain on defense-related enzyme activities in cucumber seedlings

At 20th day, the enzyme activities of LOX, PAL, CAT, PPO and SOD in cucumber seedlings treated with four treatments were significantly higher than those in CK (cucumber seedlings irrigated with sterile distilled water). Furthermore, all enzyme activities of cucumber seedlings inoculated with *Foc* after inoculation with PJH16 strain (treatment 4) were significantly higher than those of cucumber seedlings inoculated with *Foc* alone (treatment 2), with an increase of 155.68, 79.03, 71.55, 155.94 and 42.55%, respectively. However, the activities of PAL, CAT and SOD were significantly increased after inoculation with *Foc* and Hymexazol, but LOX and PPO were significantly decreased (Table 2).

**Table 2:** The enzyme activities of cucumber seedling at 20 days of treatment.

	LOX (U/g)	PAL (U/g)	CAT (U/g)	PPO (U/g)	SOD (U/g)
CK	459.85 ± 33.31^a^	11.71 ± 1.13^e^	106.08 ± 3.78^e^	22.85 ± 1.61^d^	105.21 ± 4.06^d^
*Foc*	1545.24 ± 42.43^b^	21.27 ± 2.52^c^	152.59 ± 2.75^c^	74.03 ± 4.5^b^	159.57 ± 1.46^c^
*Foc* + 0.1%Hymexazol	636.59 ± 18.05c	28.18 ± 0.88^b^	184.74 ± 7.32^b^	43.76 ± 2.62^c^	195.91 ± 11.01^b^
*Foc* + PJH16	3950.9 ± 53.91^a^	38.08 ± 4.18^a^	261.77 ± 9.43^a^	189.47 ± 6.16^a^	227.47 ± 1.07^a^
PJH16	639.21 ± 11.23^c^	19.33 ± 1.03^d^	129.70 ± 3.09^d^	71.37 ± 4.21^b^	160.41 ± 2.49^c^

The data are presented as mean ± standard deviation (SD). Within the same column, different letters (a–c) indicate significant differences at *p* < 0.05 level.

### 3.4 Cucumber seedling growth promoting activity of PJH16 strain

**Figure 3:**
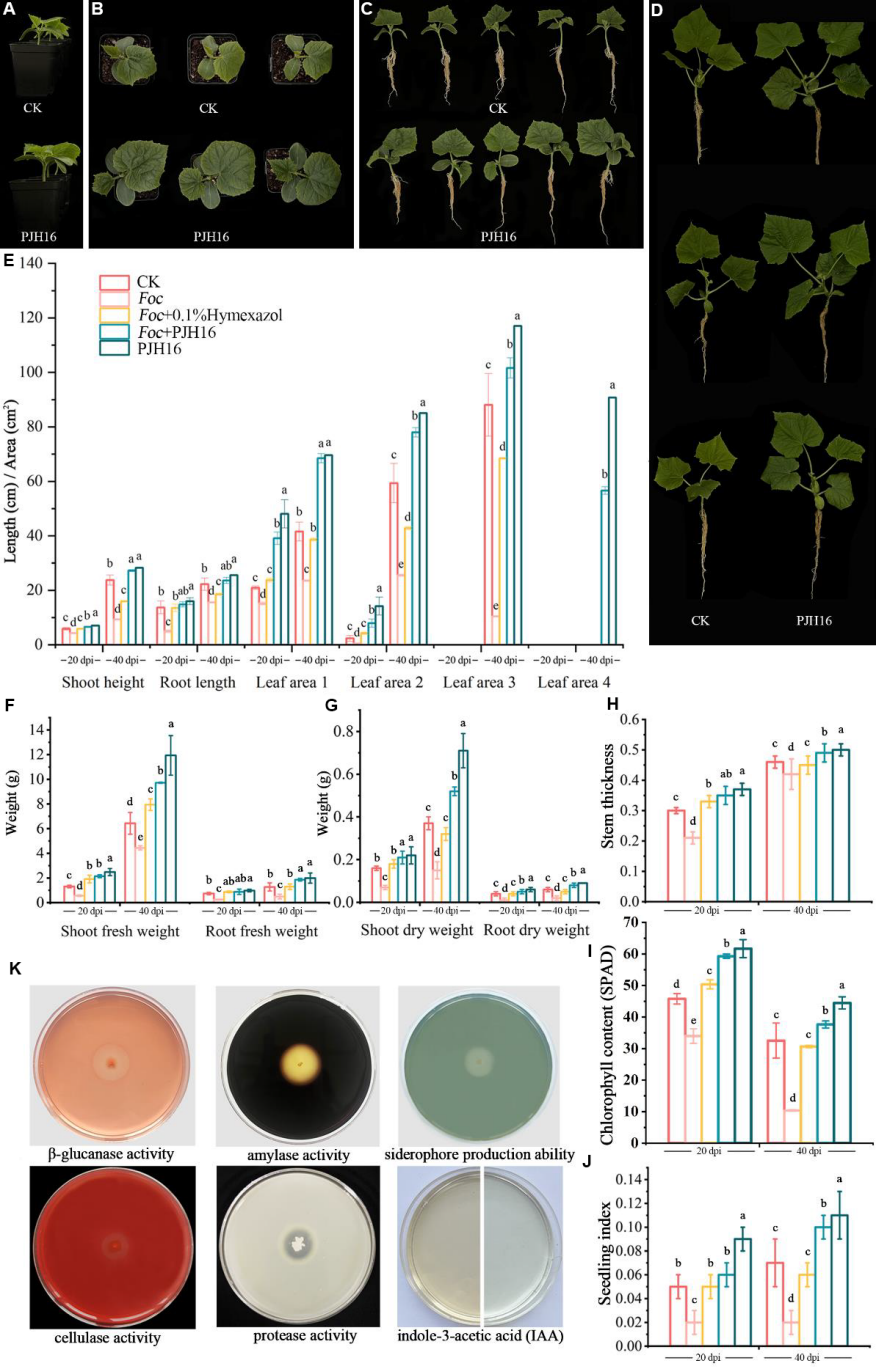
The growth-promoting effect of PJH16 strain on cucumber seedlings and its antifungal and growth-promoting properties *in vitro* were detected. **(A–C)** Control treatment (CK) and PJH16-inoculated cucumber seedlings at 20 days post-inoculation (dpi); **(D)** Control treatment (CK) and cucumber seedlings inoculated with PJH16 at 40 dpi; **(E)** Evaluation of plant height, root length, and leaf area of cucumber seedlings inoculated at 20 and 40 dpi; **(F)** Measurement of shoot fresh weight and root fresh weight for cucumber seedlings inoculated at 20 and 40 dpi; **(G)** Assessment of shoot dry weight and root dry weight for cucumber seedlings inoculated at 20 and 40 dpi; **(H)** Determination of stem thickness for cucumber seedlings inoculated at 20 and 40 dpi across five treatments; **(I)** Quantification of chlorophyll content in cucumber seedlings inoculated at 20 and 40 dpi; **(J)** Calculation of the robust seedling index for cucumber seedlings inoculated with five treatments at 20 and 40 dpi; The first row of **(K)** from left to right showcases the detection of β-glucanase activity, amylase activity, and siderophore production ability of the PJH16 strain; the second row from left to right displays the detection of cellulase activity, protease activity, and indole-3-acetic acid (IAA) production ability of the PJH16 strain. Within the same column, different letters (a–e) indicate significant differences at *p* < 0.05 level.

At 20 and 40 days, the chlorophyll content, plant height, stem diameter, root length, leaf area, shoot fresh weight and dry weight, and root fresh weight and dry weight of seedlings treated with only PJH16 strain (treatment 5) were remarkably increased (Figure 3; Supplementary Table S2). At 20 days, all indexes of treatment 5 were significantly higher than those of the control treatment. Among them compared with the control, not only the true leaf area increased the most, but also the area of the first true leaf and the second true leaf treated with treatment 5 increased by 129.63 and 503.40%, respectively (Figures 3B,C,E). As well, the fresh weight of the aboveground part increased by 88.99% (Figure 3F). Moreover, by the 40^th^ day, the most substantial increase occurred in the average leaf area of the fourth true leaf in treatment 5, reaching 90.75 cm^2^, while the leaf area of the fourth true leaf in the control treatment remained below 5 cm^2^ (Figures 3D,E). Consequently, the seedling index of the PJH16 bacterial treatment (0.06) exhibited a significant increase compared to the control (0.05) at the 20-day mark (Figure 3J). By the 40^th^ day, the seedling index for the PJH16 treatment (0.11) remained notably higher than that of the control (0.07) (Figure 3J). This outcome underscores the positive impact of the isolated bacterial strain PJH16 on enhancing plant growth.

### 3.5 Detection of antifungal and growth-promoting properties *in vitro*

The *in vitro* antifungal and growth-promoting properties of PJH16 showed that PJH16 had the activities of β-1,3-glucanase, cellulase, amylase and protease, and could act on the cell wall of fungi by secreting these enzymes to antagonize pathogens. In addition, in terms of promoting plant growth, we found that PJH16 can produce indole acetic acid and siderophores, which is beneficial to plant growth and development (Figure 3K).

### 3.6 Whole genome sequencing and assembly of PJH16 strain

**Figure 4:**
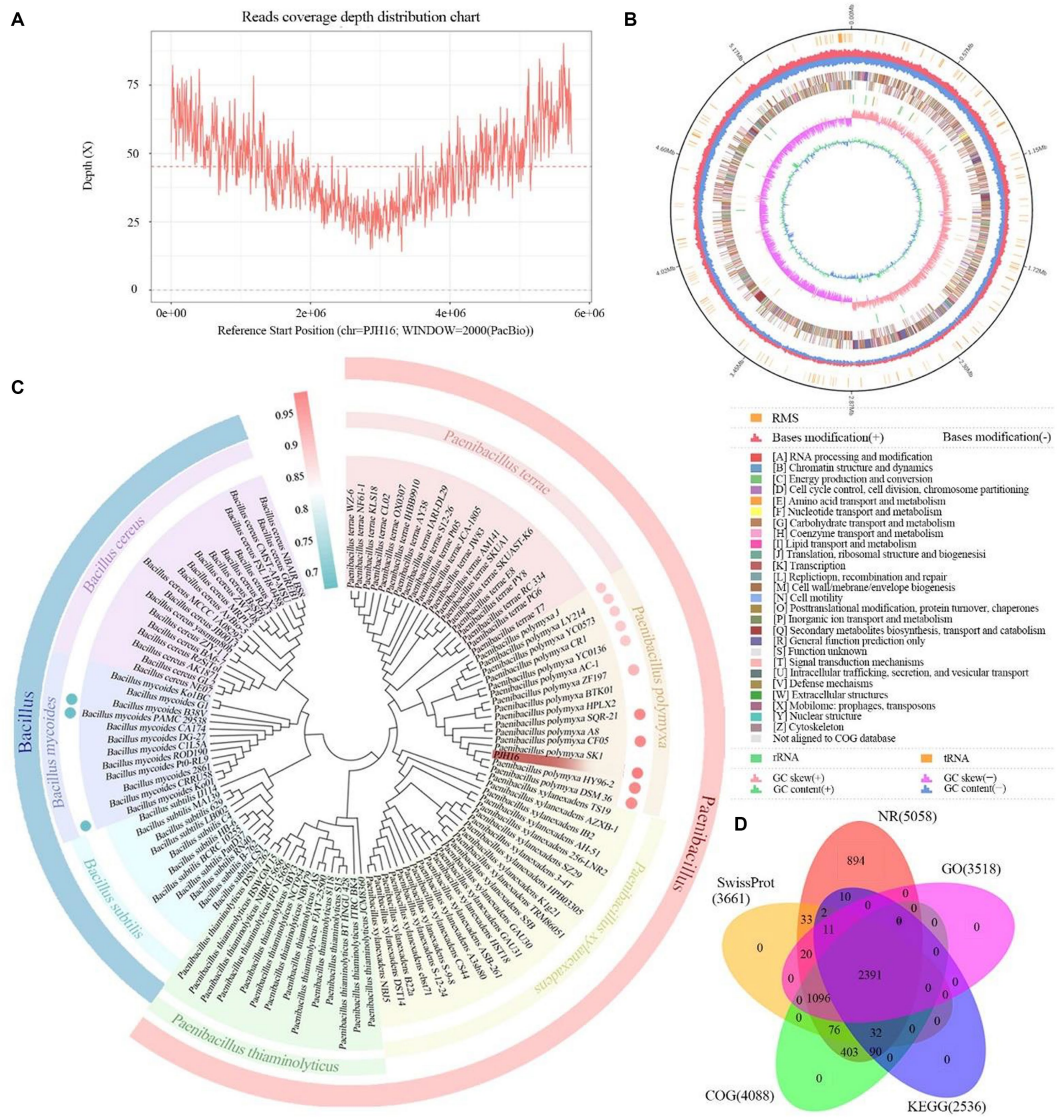
The basic information of whole genome sequencing, assembly, identification and functional annotation of *P. polymyxa* PJH16. **(A)** Visualization of genome coverage depth distribution. **(B)** Comprehensive genomic map of *P. polymyxa* PJH16. The concentric circles represent, from the innermost to the outermost: genome size (depicted by a black line); distribution of restriction modification systems on the forward (depicted in red) and reverse (depicted in blue) strands; COG classification of protein-coding genes, spanning both the forward and reverse strands; distribution of tRNA (depicted in brown) and rRNA (depicted in green); GC skewness; GC content. **(C)** Taxonomic identification of *P. polymyxa* PJH16. The concentric circles represent: Phylogenetic relationships of *P. polymyxa* PJH16 with 114 strains across 7 genera of *Bacillus* and *Paenibacillus*; Average Nucleotide Identity (ANI) analysis of *P. polymyxa* PJH16 compared to 10 strains of *P. polymyxa* and 3 strains of *B. mycoides*. **(D)** Comprehensive analysis and statistical representation of shared and unique annotations in the foundational database.

In this research endeavor, we employed a dual approach utilizing PacBio RSII to construct third-generation sequencing library. This strategy facilitated the efficient and accurate completion of whole-genome sequencing for the strains, allowing for a comprehensive exploration of their disease resistance mechanisms. The DNA sequencing of the strain yielded a total of 24,869 High Fidelity reads, characterized by an average length of 10,450.15 bp and an N50 of 10,764 bp. The genome showcased an average coverage depth of 45.18X (Figure 4A), and the assembled genome sequence has been meticulously cataloged in GenBank with the accession number CP137742. Comprising a circular chromosome spanning 5,746,138 bp, the strain’s genome exhibited a GC content of 45.81% (Figure 4D). Through analysis, a total of 5,187 protein-coding genes were predicted, encompassing 86.36% of the genome. The remaining 208 genes comprised tRNA (111 genes), rRNA (42 genes), and other non-coding RNAs (55 genes) (Figure 4B). Annotation of the protein sequences from the predicted genes against various databases (E value = 1e^−5^) unveiled matches for 5,058 (99.12%), 4,088 (76.44%), 2,536 (54.85%), and 3,518 (75.39%) protein-coding genes in NR, SwissProt, COG, KEGG, and GO databases, respectively (Figure 4D; Supplementary Table S3).

In the NR database, 53.5% of the sequences exhibited the highest similarity to species in the *Paenibacillus* group, followed by 41% of the sequences showing significant similarity to *P. polymyxa*. Additionally, 1.86% of the sequences were most similar to those of *P. polymyxa* SQR-21 (Supplementary Figure S1A). When compared with the SwissProt database, 49.17% of the sequences displayed the highest similarity to *Bacillus subtilis* (Supplementary Figure S1B).

### 3.7 Species identification and phylogenetic analysis of PJH16 strain

Based on the results of the phylogenetic tree, we found that the strain PJH16 clustered with 15 *P. polymyxa* strains, indicating that it had a close relationship with the *P. polymyxa* strain (Figure 4C). Based on ANI analysis, the gene correlation between PJH16 and 10 *P. polymyxa* strains with known whole genome sequences was analyzed. It was found that the ANI value between PJH16 and *P. polymyxa* HY96-2, *P. polymyxa* DSM36, *P. polymyxa* SQR-21 and *P. polymyxa* CF05 was >98%, which was higher than the 96% threshold (Figure 4C; Supplementary Table S4). The strain PJH16 had the highest ANI value (98.63%) with *P. polymyxa* SQR-21, and the strain PJH16 had greater homology with *P. polymyxa* SQR-21.

### 3.8 Genome function annotation of *Paenibacillus polymyxa* PJH16

Basic database annotations include NR (Supplementary Figure S1A), SwissProt (Supplementary Figure S1B), KEGG (Supplementary Figure S2A), GO (Supplementary Figure S2B), COG/KOG (Supplementary Figure S2C). Here, we use appropriate annotation tools to annotate the protein sequence of the predicted gene to the corresponding database. The whole genome of *P. polymyxa* PJH16 was annotated in the basic database to contain a large number of genes related to siderophore biosynthesis such as *feuA*, *feuB*, *feuC*, *fepC*, and *fhuC* (Supplementary Table S7).

### 3.9 Specific function database annotation

In our investigation, a comprehensive analysis of the *P. polymyxa* PJH16 genome revealed a total of 499 antibiotic resistance genes, encompassing various categories such as antibiotic synthesis genes, antibiotic resistance genes, and others, as identified through a meticulous comparison and analysis against the CARD database (Supplementary Table S6). Furthermore, a thorough annotation against the VFDB database uncovered 988 genes, featuring notable elements like *motA* and *motB*, associated with flagellar movement, *luxS*, a key quorum sensing regulatory gene, *pgaC*, involved in extracellular polysaccharide synthesis, as well as *fimB* and *fimE* genes contributing to Type 1 fimbriae formation (Supplementary Table S8).

**Figure 5:**
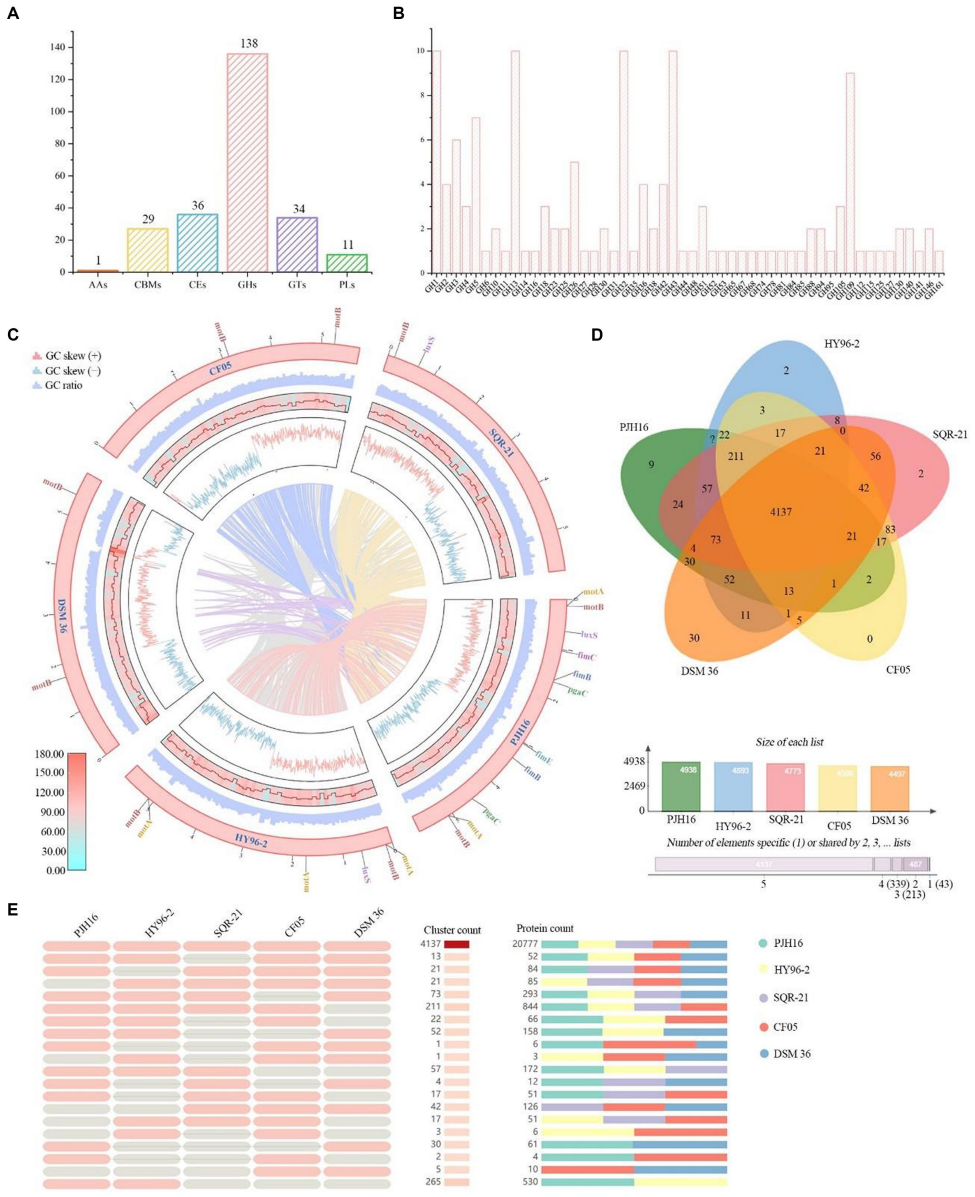
CAZymes analysis of *P. polymyxa* PJH16 genome and genome comparison of *P. polymyxa* PJH16 and four other *P. polymyxa* strains. **(A)** Distribution of gene counts across carbohydrate-active enzyme (CAZy) families in the *P. polymyxa* PJH16 genome. **(B)** Functional characterization of the glycoside hydrolase family based on the CAZy database. **(C)** Collinearity analysis of *P. polymyxa* PJH16 with three other *P. polymyxa* strains. The innermost circle depicts the comparison of *P. polymyxa* PJH16 with three other *P. polymyxa* strains, followed by the nratio, GC skews, gene density, and GC ratio of the genomes of the five *P. polymyxa* strains. Additionally, it illustrates five genes associated with biofilm formation in the *P. polymyxa* strains’ genomes. **(D)** Venn diagram showcasing the number of unique and shared gene clusters between *P. polymyxa* PJH16 and four other *P. polymyxa* strains. **(E)** Presence or absence of orthologous gene clusters in different species, where pink denotes presence and gray indicates absence.

A total of 1,390 genes in *P. polymyxa* PJH16 genome were annotated to the membrane transporter (TCDB) database, of which 532 genes were annotated to primary active transporters, 317 genes were annotated to electrochemical potential-driven transporters, and 274 genes were annotated to incompletely characterized transport systems. A total of 94 genes were annotated to channel/pores, 89 genes were annotated to group translocators, 72 genes were annotated to accessory factors involved in iransport, and 12 genes were annotated to transmembrane electron carriers (Figure 5D).

### 3.10 CAZymes analysis of *Paenibacillus polymyxa* PJH16 genome

A comprehensive analysis revealed a total of 224 genes identified as potential Carbohydrate-Active Enzymes (CAZymes), encompassing 136 Glycoside Hydrolases (GHs), 34 Glycosyl Transferases (GTs), 36 Carbohydrate Esterases (CEs), 27 Carbohydrate-Binding Modules (CBMs), 11 Polysaccharide Lyases (PLs), and 1 Auxiliary Activity (AAs) (Figure 5A). These enzymes play a pivotal role in facilitating the binding of enzymes to their substrates. Notably, 18 genes (orf00422, orf00562, orf01040, orf01052, and orf01565, etc.) were concurrently classified as GHs and CBMs, while one gene (orf00227) was categorized as CBMs and PLs simultaneously. Another gene (orf04406) was concomitantly classified as CEs and PLs. Within the GH family, antifungal CAZymes were identified, including chitinase (GH18), cellulase (GH5), endoglucanase (GH5, GH51), β-glucanase (GH1), starch debranching enzyme (GH13_31, GH13_28, GH13_29), and L-fructosyltransferase (GH32), all of which have the potential to impede the growth of plant pathogens (Figure 5B). The GH43 family exhibited a diverse range of enzymes such as α-L-arabinofuranosidase, β-D-xylosidase, α-L-arabinosidase, and β-D-galactosidase involved in the debranching and degradation of hemicellulose and pectin polymers. Additionally, the GH3 enzyme, predominantly comprised of stereochemically retained β-glucosidase, and the β-N-acetylglucosaminidase subfamily were identified. These enzymes, widely distributed in bacteria, fungi, and plants, serve multiple functions such as cellulose biomass degradation, plant and bacterial cell wall remodeling, energy metabolism, and defense against pathogens. The genomic distribution of CAZymes in strain PJH16 underscores its potential to counteract fungal pathogens.

### 3.11 Secondary metabolic potential of *Paenibacillus polymyxa* PJH16

Within the genomic landscape of *P. polymyxa* PJH16, a total of 19 gene clusters associated with the biosynthesis of secondary metabolites were discerned (Table 3). These putative gene clusters are diverse, encompassing 9 clusters dedicated to the encoding of NRPS (nonribosomal peptide synthetase), 3 clusters for transAT-PKS (trans-acyltransferase polyketide synthase), 4 clusters affiliated with lanthipeptide-class-i, 3 clusters for cyclic-lactone-autoinducer, 1 cluster each for RRE-containing, proteusin, lassopeptide, T1PKS, betalactone, and ranthipeptide. Notably, Cluster 4 and Cluster 6 have the capability to encode both NRPS and transAT-PKS, while Cluster 9 encodes both NRPS-like and cyclic-lactone-autoinducer. Cluster 12 can encode both T1PKS and NRPS. Cluster 15 encodes transAT-PKS, NRPS, T3PKS and PKS-like. Among the nine clusters encoding NRPS, four clusters had 100% similarity to the known fusaricidin B, tridecaptin and polymyxin synthetic clusters, respectively, and the clusters encoding T1PKS and NRPS had 5% similarity to the laterocidine synthetic cluster. The cluster that encodes transAT-PKS, NRPS, T3PKS and PKS-like has 35% similarity with aurantinin B/aurantinin C/aurantinin D synthetic cluster. The four clusters encoding lanthipeptide-class-i are 100% similar to known synthetic clusters of paenibacillin, paenilan, S-layer glycan, and paenicidin A, respectively. The cluster similar to the paenibacillin synthetic cluster also encodes cyclic-lactone-autoinducer. Within the antiSMASH database, nine distinct biosynthetic gene clusters were identified, exhibiting dissimilarity to known clusters. These novel gene clusters appear to be potential candidates for the biosynthesis of secondary metabolites, necessitating further isolation and characterization. Notably, two of these clusters are implicated in transAT-PKS and NRPS biosynthesis, while one cluster is associated with the synthesis of NRPS-like and cyclic-lactone-autoinducer. The remaining clusters encompass a variety of functionalities, including RRE-containing, proteusin, Lassopeptide, NRPS, cyclic-lactone-autoinducer, and ranthipeptide. It’s noteworthy that the 19 gene clusters responsible for secondary metabolite biosynthesis in the genome of *P. polymyxa* PJH16 were also observed in the genomes of the other four *P. polymyxa* strains. However, the specific composition of gene clusters in *P. polymyxa* PJH16 differs from each of the three other strains (Supplementary Table S9).

**Table 3:** The hypothetical gene cluster lists encoding secondary metabolites predicted by antiSMASH extracted from genomes of *P. polymyxa* PJH16.

Clusters	Types	Start	End	Most similar known clusters	Similarity
Clusters 1	NRPS	68,307	131,002	Fusaricidin B	100%
Clusters 2	RRE-containing	359,180	379,398		
Clusters 3	Lanthipeptide-class-i, cyclic-lactone-autoinducer	1,008,996	1,034,702	Paenibacillin	100%
Clusters 4	transAT-PKS, NRPS	1,051,529	1,127,741		
Clusters 5	Proteusin	1,240,271	1,260,507		
Clusters 6	NRPS, transAT-PKS	1,293,688	1,392,182		
Clusters 7	Lassopeptide	1,428,174	1,452,244		
Clusters 8	Lanthipeptide-class-i	1,749,619	1,774,887	Paenilan	100%
Clusters 9	NRPS-like, cyclic-lactone-autoinducer	2,044,764	2,105,850		
Clusters 10	NRPS	2,469,722	2,563,254	Tridecaptin	100%
Clusters 11	NRPS	2,720,413	2,801,157		
Clusters 12	T1PKS, NRPS	2,807,997	2,882,188	Laterocidine	5%
Clusters 13	Betalactone	2,969,594	3,000,473	Thermoactinoamide	100%
Clusters 14	Cyclic-lactone-autoinducer	3,041,510	3,062,058		
Clusters 15	transAT-PKS, NRPS, T3PKS, PKS-like	3,566,941	3,668,855	Aurantinin B/aurantinin C/aurantinin D	35%
Clusters 16	Ranthipeptide	4,369,396	4,394,783		
Clusters 17	NRPS	4,885,551	4,965,075	Polymyxin	100%
Clusters 18	Lanthipeptide-class-i	5,058,332	5,080,559	S-layer glycan	28%
Clusters 19	Lanthipeptide-class-i	5,415,752	5,442,186	Paenicidin A	100%

### 3.12 Comparison of genomes

In order to obtain the collinearity analysis between the *P. polymyxa* PJH16 genome and the other four *P. polymyxa* strains, the whole genome sequences (*P. polymyxa* HY96-2, *P. polymyxa* SQR-21, *P. polymyxa* CF05 and *P. polymyxa* DSM 36) in the NCBI sequence library were analyzed for collinearity (Figure 5C). The results showed that *P. polymyxa* PJH16 had the best collinearity with *P. polymyxa* HY96-2 and *P. polymyxa* SQR-21, and the genome structure arrangement was basically similar without gene mutation, which proved the close relationship between the two strains and supported our phylogenetic results. There was strong chances Horizontal gene transfer (HGT) between *P. polymyxa* PJH16 strain and *P. polymyxa* CF05 and *P. polymyxa* DSM 36, respectively (Figure 5C). At the same time, we also annotated the genes related to biofilm formation in these five strains. We found that the genes related to biofilm formation were the most annotated in the genome of *P. polymyxa* PJH16.

We also performed pan-genome analysis to compare *P. polymyxa* PJH16 with these four *P. polymyxa* strains. As shown in the figure, the number of homologous genes of *P. polymyxa* PJH16, *P. polymyxa* HY96-2, *P. polymyxa* SQR-21, *P. polymyxa* CF05 and *P. polymyxa* DSM 36 were 4,938, 4,893, 4,773, 4,596, and 4,497, respectively. The number of single copy genes was 205, 261, 225, 71, and 549, respectively (Table 4). The number of homologous genes shared by the four strains was 4,137, and there were 9 unique gene clusters of *P. polymyxa* PJH16. There were 2 unique gene clusters in *P. polymyxa* HY96-2. There are 2 unique gene clusters in *P. polymyxa* SQR-21; there are 30 specific gene clusters in *P. polymyxa* DSM 36; *P. polymyxa* CF05 had no specific gene cluster (Figures 5D,E).

**Table 4:** The number of proteins, clusters, and singletons in *P. polymyxa* PJH16 and four other *P. polymyxa* strains.

Species	Proteins	Clusters	Singletons
PJH16	5,187	4,938	205
HY96-2	5,180	4,893	261
SQR-21	5,024	4,773	225
CF05	4,685	4,596	71
DSM 36	5,096	4,497	549

## 4 Discussion

In our study, it was examined the effect of bacterial strain PJH16 on the disease *Fusarium oxysporum* f. sp. *cucumerinum* both *in vitro* and *in vivo*, and followed by investigating the mechanism of illness biological control by using the molecular identification of the bacterial strain PJH16 to provide information for efficient *Fusarium* wilt disease management in cucumber.

This research began with the isolation of *P. polymyxa* PJH16 strain from the rhizosphere soil of healthy cucumber that was verified by double culture experiments to check the effective inhibition for the *Fusarium oxysporum* f. sp. *cucumerinum*, and its antagonistic band width that reached 11 mm. The obtained results showed that the genome comprises 110450.15 bp circular chromosome, and its GC content is 45.81%.

When defining a species, the phylogenetic theory considers the evolutionary and genealogical relationships between organisms. Thus, the phylogenetic tree analysis based on 16S rRNA gene sequence achieved that the strain PJH16 had a close relationship with *P. polymyxa* genus and the *P. polymyxa* HY96-2 was the closest, followed by *P. polymyxa* DSM 36 strain. According to ANI analysis, PJH16 was the most similar to *P. polymyxa* HY96-2, followed by *P. polymyxa* SQR-21, *P. polymyxa* DSM 36 and *P. polymyxa* CF05. This analysis indicated that PJH16 strain belongs to *P. polymyxa* genera, additionally is closely related to *P. polymyxa* HY96-2, *P. polymyxa* SQR-21, *P. polymyxa* DSM 36 and *P. polymyxa* CF05. In such a case, horizontal gene transfer (HGT) plays a vital role in the structure of bacterial genomes and provides the raw material for natural selection in diversifying bacterial lineages, especially in the evolution of specific lineages, species and subspecies (Nowell et al., 2014). Through the global alignment of *P. polymyxa* PJH16, *P. polymyxa* SQR-21, *P. polymyxa* CF05 and *P. polymyxa* DSM 36 chromosomes by Mauve and TBtools visualization, we found that the genome of *P. polymyxa* PJH16 was highly homologous to *P. polymyxa* HY96-2 and *P. polymyxa* SQR-21. At the same time, collinearity analysis showed that there may be horizontal gene transfer between *P. polymyxa* PJH16 and the other two strains. In addition, genome comparative analysis showed that *P. polymyxa* PJH16 had the largest number of encoded proteins and gene clusters. Such results indicate that *P. polymyxa* PJH16 has a certain specificity, and its biological control mechanism may differ from that of other *P. polymyxa*.

Many bacteria belonging to *P. polymyxa* have the ability to promote plant growth and inhibit plant pathogens. They have potential plant growth promoting ability and known as Plant Growth Promoting Rhizobacteria (PGPR) (Timmusk and Wagner, 1999) and biological control agents (BCAs) (Lee et al., 2020). Despite this strain is widely distributed naturally, has a wide rage of industrial, agricultural, and environmental applications, and is generally regarded as an environmentally safe bacterium (Timmusk and Wagner, 1999), there have been few reports on the analysis of *P. polymyxa* gene sequence to date. Therefore, it was important to understand the biological control mechanism of *P. polymyxa* PJH16 at the molecular level. The predicted mechanisms are biofilm formation, the production of antifungal compounds, resistance induction in plants, and plant growth promotion.

**Figure 6:**
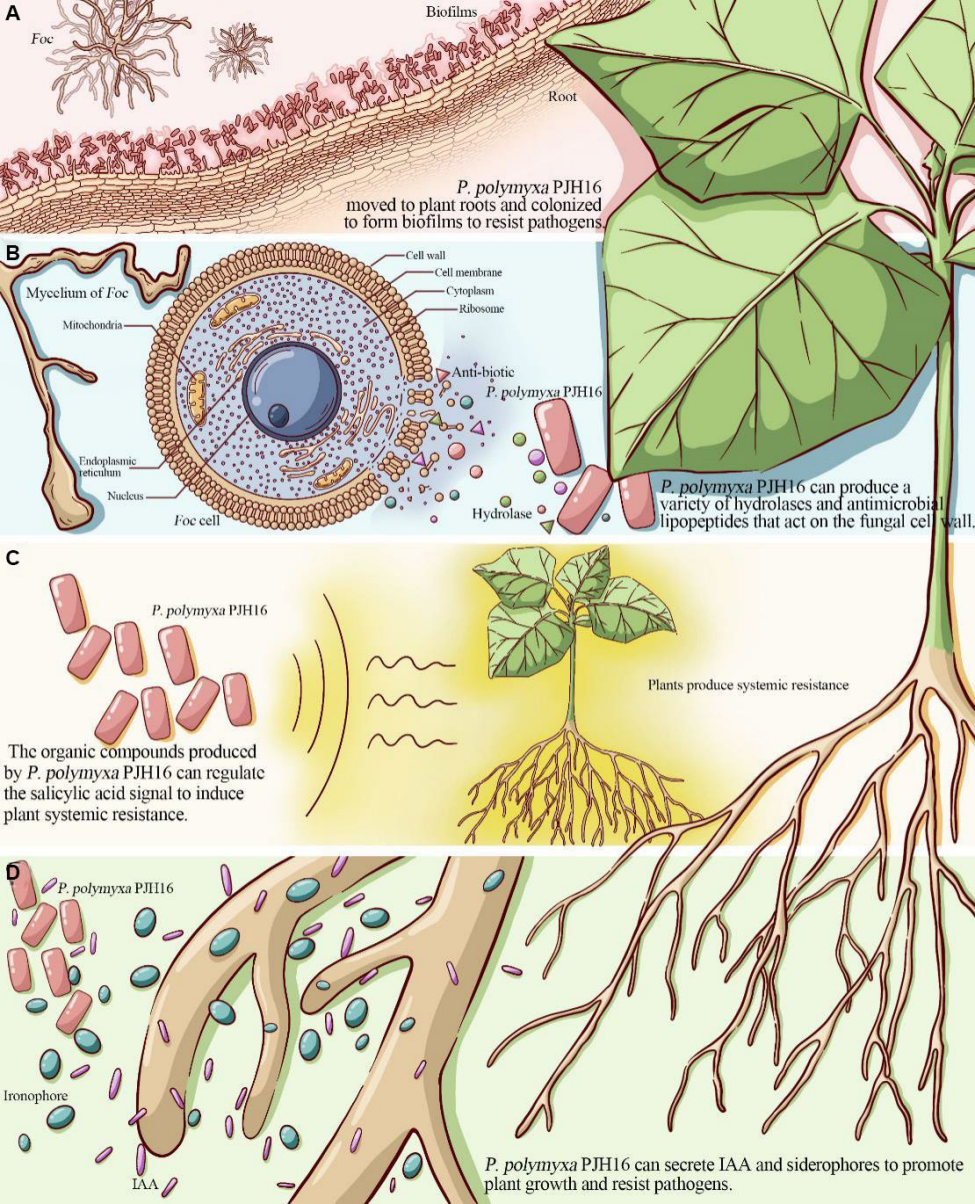
The proposed mechanism of *P. polymyxa* PJH16 antagonizing Foc. (A) *P. polymyxa* PJH16 moved to plant roots and colonized to form biofilms to resist pathogens. (B) *P. polymyxa* PJH16 can produce a variety of hydrolases and antimicrobial lipopeptides that act on the fungal cell wall. (C) The organic compounds produced by *P. polymyxa* PJH16 can regulate the salicylic acid signal to induce plant systemic resistance. (D) *P. polymyxa* PJH16 can secrete IAA and siderophores to promote plant growth and resist pathogens.

Biofilm formation helps bacteria establish self-protection mechanisms, affects the bacterial response to nutrients in the environment and antibiotic resistance, and promotes root colonization (Ongena and Jacques, 2008). For instance, the isolated *P. polymyxa* B1 and B2 strains can colonize in the intercellular space after invading the roots of *Arabidopsis thaliana*, and then form a biological protective film on its surface, which can effectively prevent pathogenic fungus from invading *Arabidopsis thaliana* (Timmusk et al., 2005). With a deeper understanding of genetic identification, the key genes affecting biofilm formation included flagellar movement-related genes, quorum sensing genes, and extracellular polysaccharide production-related genes. The *motA* and *motB* genes related to the formation and movement of flagella (Platzer et al., 1997), were found in the genome of PJH16. In the context of biofilm formation, the quorum sensing (QS) system plays a pivotal role in orchestrating gene expression and coordinating cellular activities. This regulatory mechanism is recognized as a significant factor in governing biofilm development across various bacterial species (Miller and Bassler, 2001; Raafat et al., 2019). Notably, our genomic analysis revealed the presence of the *luxS* gene in the PJH16 strain. *LuxS* is a pivotal regulatory gene within the quorum sensing system, specifically mediated by autoinducer 2 (AI-2) (Ma et al., 2017). Another gene involved in biofilm formation in PJH16 is *pgaC* gene, which is involved in the synthesis of polymer poly β-1,6-N-acetyl-D-glucosamine (PNAG) (Chen et al., 2014). The type 1 fimbriae regulated by *fimB* and *fimE* genes can help *P. polymyxa* to bio-attach along the roots in the soil, positively impacting plant growth and health. From the above results, it can be speculated that the PJH16 strain can hinder the attachment and invasion of fungal pathogens by forming a tightly bound biofilm, and seize the nutrition and growth space required by fungi. In addition, extracellular polysaccharides and antifungal substances can interfere with the key processes of fungal cell wall synthesis, nutrient uptake and metabolism, thereby inhibiting the reproduction and spread of fungal pathogens (Figure 6A). However, whether *P. polymyxa* PJH16 can produce biofilm still needs to be verified by relevant experiments.

One of the biological control mechanisms is to secrete antagonistic substances for inhibiting fungal disease (XueLi et al., 2008). According to their material types, they can be divided into hydrolases (Beatty and Jensen, 2002) such as protease, β-1,3-glucanase, cellulase, xylanase and chitinase (Kwon et al., 2016), polypeptide antibiotics (Raza et al., 2008) such as polymyxin, fusaricidin and colistin (Naghmouchi et al., 2012), and plant hormones (Niu et al., 2013) such as cytokinin, auxin and indole phenol (Mei et al., 2014; Park et al., 2014). Previously, it was shown that *P. polymyxa* can inhibit spore germination and mycelial growth of Fusarium by producing glucanase, chitinase, cellulase and protease related to the destruction of eukaryotic cell wall (Nielsen and Sorensen, 1997; Huang et al., 2020). By employing scanning electron microscopy approach to observe the mycelium of *Foc* in the dual culture antagonism experiment, the *P. polymyxa* PJH16 strain caused deformation of the mycelium, as twisting, folding and shrinkage. Simultaneously, *in vitro* antifungal properties test showed that *P. polymyxa* PJH16 could secrete protease, amylase, β-glucanase and cellulase. These findings pointed out that there is a role of *P. polymyxa* PJH16 strain in inhibiting the growth and development of *F. oxysporum* by producing substances such as hydrolases and antibiotics. Furthermore, the genome of *P. polymyxa* PJH16 has the synthetic gene clusters of fusaricidin and paenibacillin, which have inhibitory effects on fungi. Nine gene clusters in the genome of *P. polymyxa* PJH16 have not been found to have homologous proteins in the database. Among them, ranthipeptides are free radical non-α-sulfur-containing peptides, which are a new class of natural products and belong to the ribosome synthesis and post-translational modification peptide (RiPP) superfamily. Studies have shown that ranthipeptides may be involved in quorum sensing and control of cell metabolism (Chen Y. L. et al., 2020). Similar to cyclic peptides, cyclic-lactone-autoinducer is a class of compounds that play a role in intercellular communication in bacteria. By binding to specific receptor proteins, it triggers signal transduction pathways within bacteria and regulates bacterial growth, development, biofilm formation, biological attachment, and toxin production (Mull et al., 2018). Whereas, *P. polymyxa* SQR-21 and *P. polymyxa* CF05 lacked the synthetic gene cluster of paenibacillin. Taken together, the differences in antibiotic synthesis gene clusters between *P. polymyxa* PJH16 and other *P. polymyxa* may lead to differences in their ability to control plant diseases (Figure 6B). However, this is only based on the prediction of the genome of *P. polymyxa* PJH16, and does not specifically detect whether the strain can produce related compounds. Therefore, subsequent experiments are needed to verify its metabolites.

The results of the existing research on the mechanism of biocontrol bacteria confirmed that some biocontrol bacteria have induced systemic resistance (ISR) in plants under the ‘plant-pathogen-growth-promoting bacteria’ interaction mode, and can be used as non-pathogenic microorganisms to induce host plants to produce a series of systemic resistance, thereby assisting host crops to defend against pathogenic microorganisms (Qiao et al., 2017; Wang et al., 2017). ISR regulated by jasmonic acid and ethylene signaling pathways is one of the important mechanisms for the prevention and control of plant pathogen infection by plant growth promoting rhizobacteria (Pieterse et al., 2014). Antagonistic bacteria *P. polymyxa* W3 suspension and its filtrate can induce systemic resistance of tomato leaves to *Botrytis cinerea*. The activities of PAL, peroxidase POD, PPO and SOD in leaves were significantly enhanced after *P. polymyxa* W3 and its filtrate treatment (Tong et al., 2004). The current study found that inoculation of *P. polymyxa* PJH16 strain significantly increased the activities of LOX, PAL, CAT, PPO and SOD in cucumber seedlings, indicates that the strain can help cucumber plants resist *Fusarium oxysporum* infection through ISR. In addition, many previous studies have shown that fusaricidin can protect plants against pathogens through ISR. Fusaricidin produced by *P. polymyxa* WLY78 strain can induce systemic resistance to fusarium wilt of cucumber through salicylic acid (SA) signal, and significantly inhibit the production of disease (Li and Chen, 2019). Hence, we speculated that the isolated strain *P. polymyxa* PJH16 can regulate salicylic acid signals by producing fusaricidin and other compounds to make cucumbers induce systemic resistance. Such findings need to be further explored (Figure 6C).

As an important PGPR resource, *P. polymyxa* can produce auxin analogs, ethylene, indole acetic acid, cytokinins and siderophores to directly promote plant growth. *P. polymyxa* SQR-21 is not only an effective biocontrol agent against watermelon fusarium wilt, but also a plant growth-promoting rhizobacteria. It can promote the growth and development of watermelon plants by inducing the expression of several proteins involved in growth, photosynthesis and other metabolic and physiological activities (Yaoyao et al., 2017). It has been proved that the fermentation broth and bacterial suspension of *P. polymyxa* CF05 strain have growth promoting effect on tomato seedlings. The fresh weight of tomato seedlings treated with *P. polymyxa* CF05 fermentation broth increased by 272.0%, and the dry weight increased by 266.7% (Guo et al., 2014). Likewise, the growth-promoting test of cucumber seedlings verified that *P. polymyxa* PJH16 affected growth-promotion and could secrete indoleacetic acid and siderophore. It is well known that indole-3-acetic acid (IAA) is an essential plant hormone for plant growth. The competition of siderophores for iron is an important feature of many bacterial biocontrol agents against plant pathogenic fungi. Iron is one of the key elements necessary for plant growth and development, and is essential for normal metabolism and biochemical reactions of plants. Along with the same effect, an appropriate amount of iron helps to enhance the stress resistance of plants, consequently, the plants can better cope with environmental stress, such as salt stress, drought and cold. However, iron in soil is not always easily absorbed by plants. The siderophores secreted by bacteria can effectively convert insoluble iron in soil into soluble forms, making plants more easily absorbed, thus helping to prevent leaf yellowing and wilting caused by iron deficiency. Many biocontrol bacteria have the ability to produce siderophores, such as *P. polymyxa* (Padda et al., 2017), *P. riograndensis* (Beneduzi et al., 2010), *P. sinopodophylli* (Chen et al., 2016) and *P. panacihumi* (Kim et al., 2018). At the same time, the genome analysis of PJH16 showed that it contained a large number of genes related to siderophore biosynthesis, such as *feuA*, *feuB*, *feuC*, *fepC*, and *fhuC*. A broadly similar point has been confirmed that PJH16 can promote plant growth by secreting indole acetic acid and siderophores, which in turn helps plants resist pathogens (Figure 6D).

## 5 Conclusion

This study supported the idea that the *P. polymyxa* PJH16 strain can be an eco-biopesticide agent demonstrating multifunctional positive features. The phenotypic, phylogenetic, and genomic analyses have elucidated the mechanism of *P. polymyxa* PJH16 antagonizing *Fusarium* cucumber disease (Figure 6). Several factors could explain this mechanism. Firstly, *P. polymyxa* PJH16 moved to plant roots and colonized to form biofilms to resist pathogens and protect plants. Secondly, *P. polymyxa* PJH16 can produce a variety of hydrolases and antimicrobial lipopeptides that act on the fungal cell wall, such as glucanase, proteinase and cellulase; therefore, it can directly inhibit the growth of pathogenic fungi. Thirdly, the organic compounds produced by *P. polymyxa* PJH16 can regulate the salicylic acid signal to induce plant systemic resistance. Finally, *P. polymyxa* PJH16, as an important PGPR, can secrete IAA and siderophores to promote plant growth and resist pathogens. However, the two conclusions of the formation of biofilm and the production of antifungal compounds based on genome-wide analysis have not been experimentally verified, and related experiments will be carried out in the future. Although this strain has not been used in different environmental conditions, plants and pathogenic fungi to detect its broad-spectrum antifungal activity, it can be used as an ideal strain for biological control and plant growth promotion. We will also carry out relevant broad-spectrum experiments to verify whether it can be widely used in different regions.

## Data availability statement

The datasets presented in this study can be found in online repositories. The names of the repository/repositories and accession number(s) can be found in the article/Supplementary material.

## Author contributions

FY: Conceptualization, Investigation, Writing – original draft, Writing – review & editing. HJ: Data curation, Formal analysis, Methodology, Writing – original draft, Writing – review & editing. KM: Funding acquisition, Writing – review & editing. AH: Writing – review & editing. XW: Supervision, Writing – review & editing. SL: Resources, Validation, Writing – review & editing. GC: Formal analysis, Project administration, Writing – review & editing. LY: Validation, Visualization, Writing – review & editing. BT: Resources, Supervision, Writing – review & editing. XS: Resources, Writing – review & editing.
