# Supplementary figures and images for: Genomic and phenotypic analyses reveal Paenibacillus polymyxa PJH16 is a potential biocontrol agent against cucumber fusarium wilt
Source: Front Microbiol. 2024 Mar 25;15:1359263. doi: 10.3389/fmicb.2024.1359263 (PMC11000672; doi:10.3389/fmicb.2024.1359263)

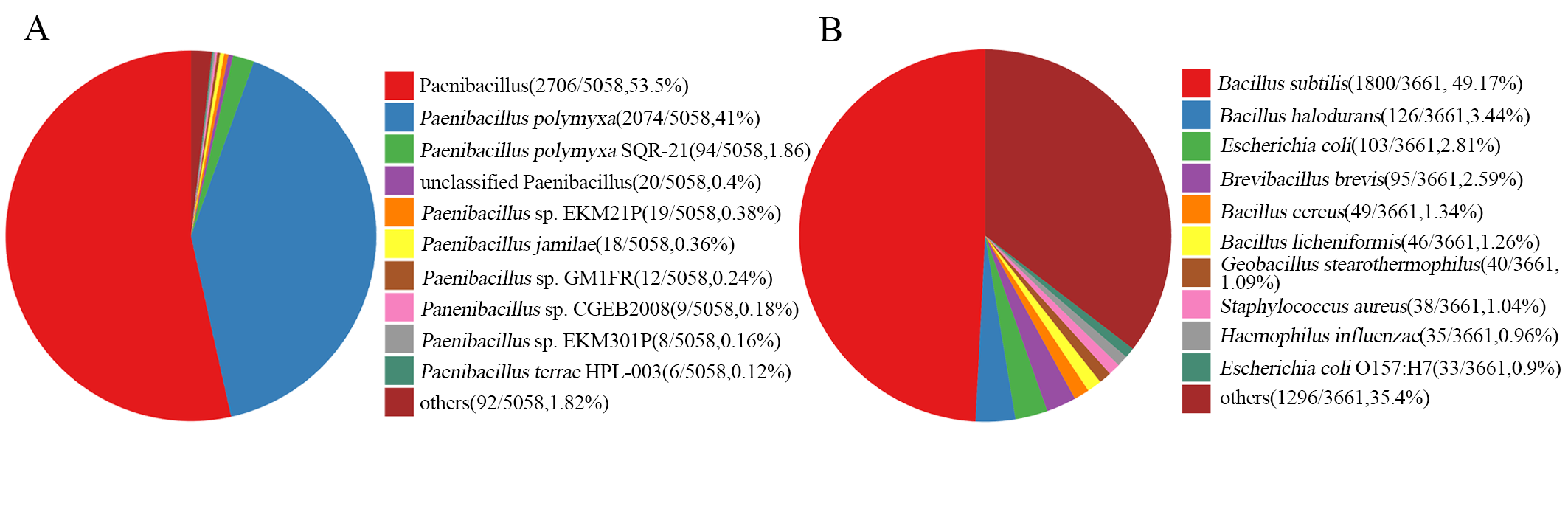

Supplement: Supplementary file 10 [file Image_1.tif]

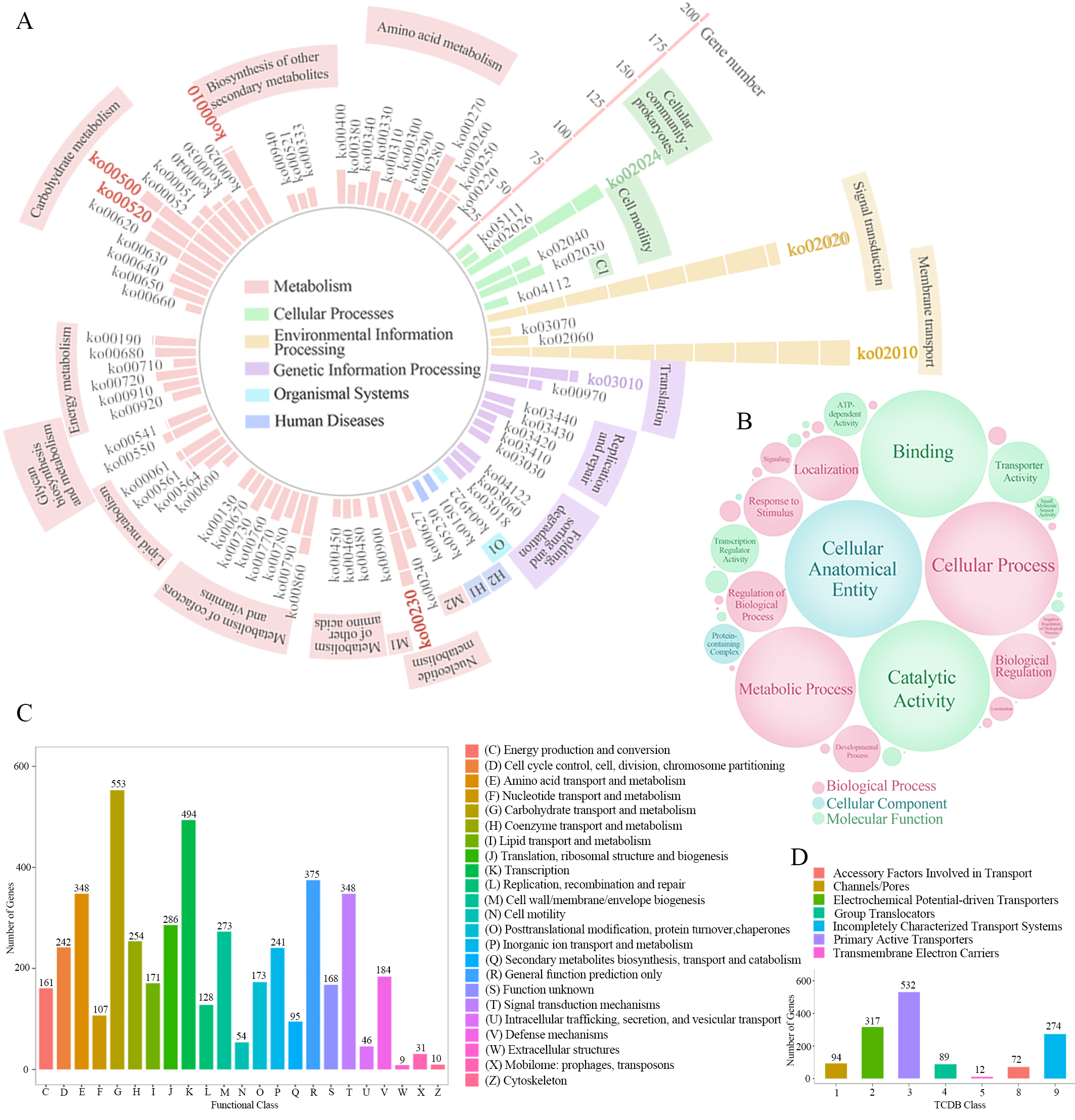

Supplement: Supplementary file 11 [file Image_2.tif]
